# Supplementary material for: Metabolic and developmental rate divergence between serially homologous cells underlies an evolutionary innovation in Drosophila
Source: PLoS Biol. 2026 Aug 24;24(8):e3003964. doi: 10.1371/journal.pbio.3003964 (PMC13502693; doi:10.1371/journal.pbio.3003964)
Supplement: Supplementary file 1 [file pbio.3003964.s001.pdf]

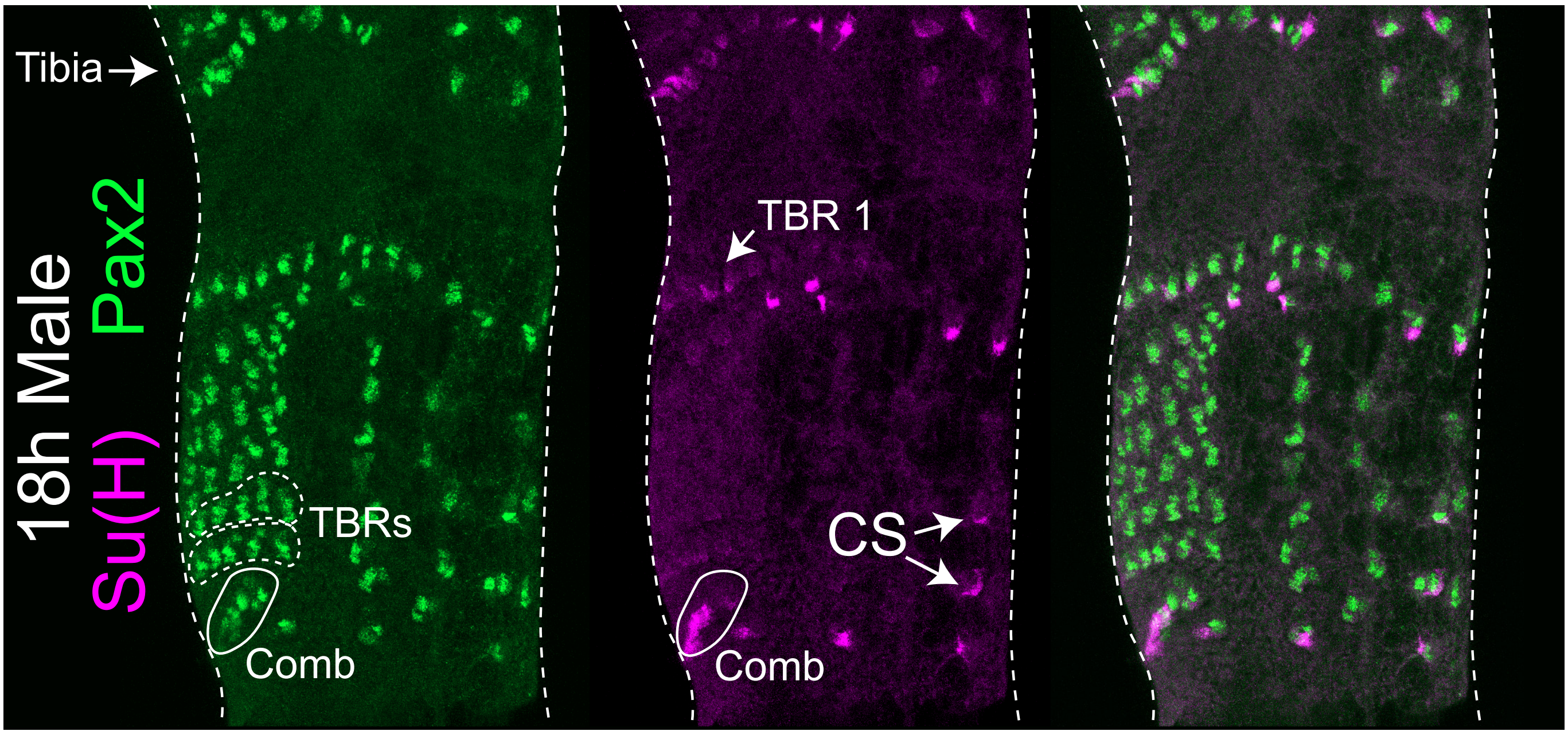

Supplementary figure 1. Sex comb cells differentiate earlier than mechanosensory bristle cells. Stainings of an 18h APF male first tarsal segment with antibodies against Pax2 and Su(H). Whereas Fig. 1D prioritizes the view of the sex comb, this image prioritizes the view of the transverse bristle rows (TBRs). CS=chemosensory bristles.
