# Supplementary material for: Metabolic and developmental rate divergence between serially homologous cells underlies an evolutionary innovation in Drosophila
Source: PLoS Biol. 2026 Aug 24;24(8):e3003964. doi: 10.1371/journal.pbio.3003964 (PMC13502693; doi:10.1371/journal.pbio.3003964)
Supplement: Supplementary file 2 [file pbio.3003964.s002.pdf]

A

Model Comparison: Likelihood Ratio Test								
Model	npar	AIC	BIC	logLik	deviance	Chisq	Df	Pr(>Chisq)
Length ~ Class + Age + (1 | LegID/BristleID)	7.000	3798.151	3836.770	−1892.075	3784.151			
Length ~ Class * Age + (1 | LegID/BristleID)	9.000	3750.542	3800.195	−1866.271	3732.542	51.609	2.000	<0.0001

B

Type III Analysis of Variance Table with Satterthwaite's Method						
Fixed effects	Sum Sq	Mean Sq	NumDF	DenDF	F value	Pr(>F)
Class	127.272	127.272	1.000	587.222	1154.546	<0.0001
Age	65.635	32.817	2.000	68.731	297.702	<0.0001
Class:Age	5.892	2.946	2.000	587.191	26.725	<0.0001

C

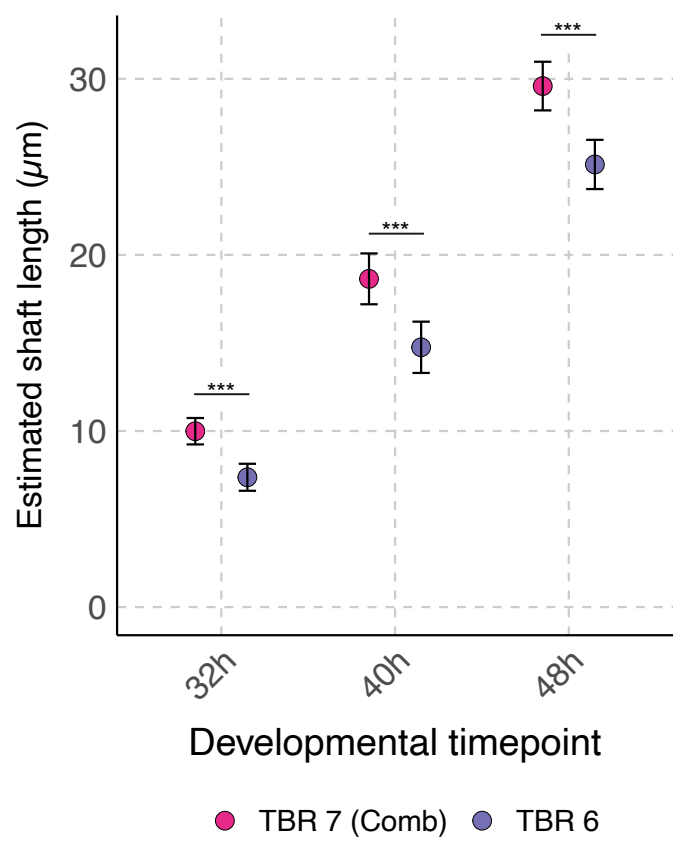

E

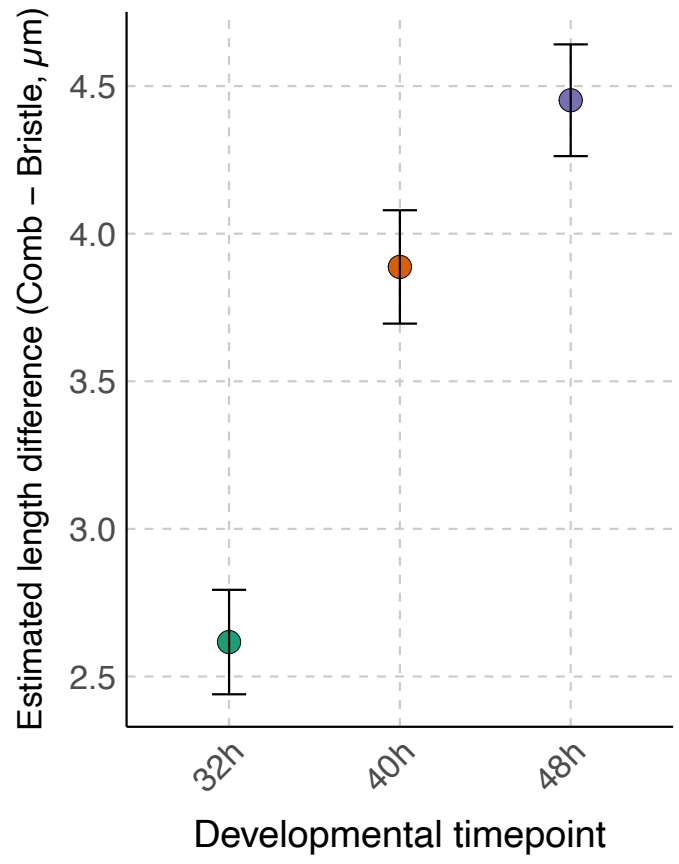

D

Estimated Shaft Lengths by Age and Bristle Row					
	Est. mean	SE	df	Lower CL	Upper CL
32h					
TBR 7 (Comb)	9.987	0.377	81.113	9.237	10.737
TBR 6	7.370	0.386	89.177	6.603	8.137
40h					
TBR 7 (Comb)	18.640	0.721	56.376	17.197	20.084
TBR 6	14.753	0.727	58.543	13.297	16.209
48h					
TBR 7 (Comb)	29.589	0.689	56.213	28.208	30.970
TBR 6	25.137	0.698	59.209	23.740	26.535

F

Model Estimates						
contrast	Age	estimate	SE	df	t.ratio	p.value
TBR 7 (Comb) - TBR 6	32h	2.617	0.177	605.787	14.789	<0.0001
TBR 7 (Comb) - TBR 6	40h	3.887	0.192	570.030	20.239	<0.0001
TBR 7 (Comb) - TBR 6	48h	4.452	0.189	570.052	23.520	<0.0001

Supplementary figure 2. Shaft growth is accelerated in the sex comb relative to the surrounding mechanosensory bristles. (A) The output of a likelihood ratio test comparing two linear mixed effects models that model the effect of organ 'class' (sex comb or mechanosensory bristle) and developmental stage ('Age': 32h, 40h, and 48h after puparium formation) on shaft length. One model includes the interaction term while the other omits it. As random effects, we include the three length measurements taken per individual bristle ('BristleID') nested within an individual leg ('LegID'). The significant p-value ($p < 0.0001$) indicates that the inclusion of the interaction term significantly improves model fit and, therefore, that the difference in shaft length between the sex comb teeth and mechanosensory (MS) bristles increases with age. (B) F-values and p-values from the full model specified in (A), i.e., the interaction term model. (C) Estimated marginal means and confidence limits derived from the full model using the R package 'emmeans'. (D) The estimated marginal means plotted in (C). (E) The difference in estimated marginal mean lengths between teeth in the comb (i.e., TBR 7) and MS bristles (TBR 6). (F) Within-timepoint contrasts conducted on the emmeans output using the function 'pairs' and a Tukey p-value adjustment. The contrast estimates are plotted in (E) and the p-values correspond to those plotted in (C). The data and code needed to generate the plots in this Figure can be found in https://osf.io/25bnc.
