# Supplementary material for: Metabolic and developmental rate divergence between serially homologous cells underlies an evolutionary innovation in Drosophila
Source: PLoS Biol. 2026 Aug 24;24(8):e3003964. doi: 10.1371/journal.pbio.3003964 (PMC13502693; doi:10.1371/journal.pbio.3003964)
Supplement: Supplementary file 5 [file pbio.3003964.s005.pdf]

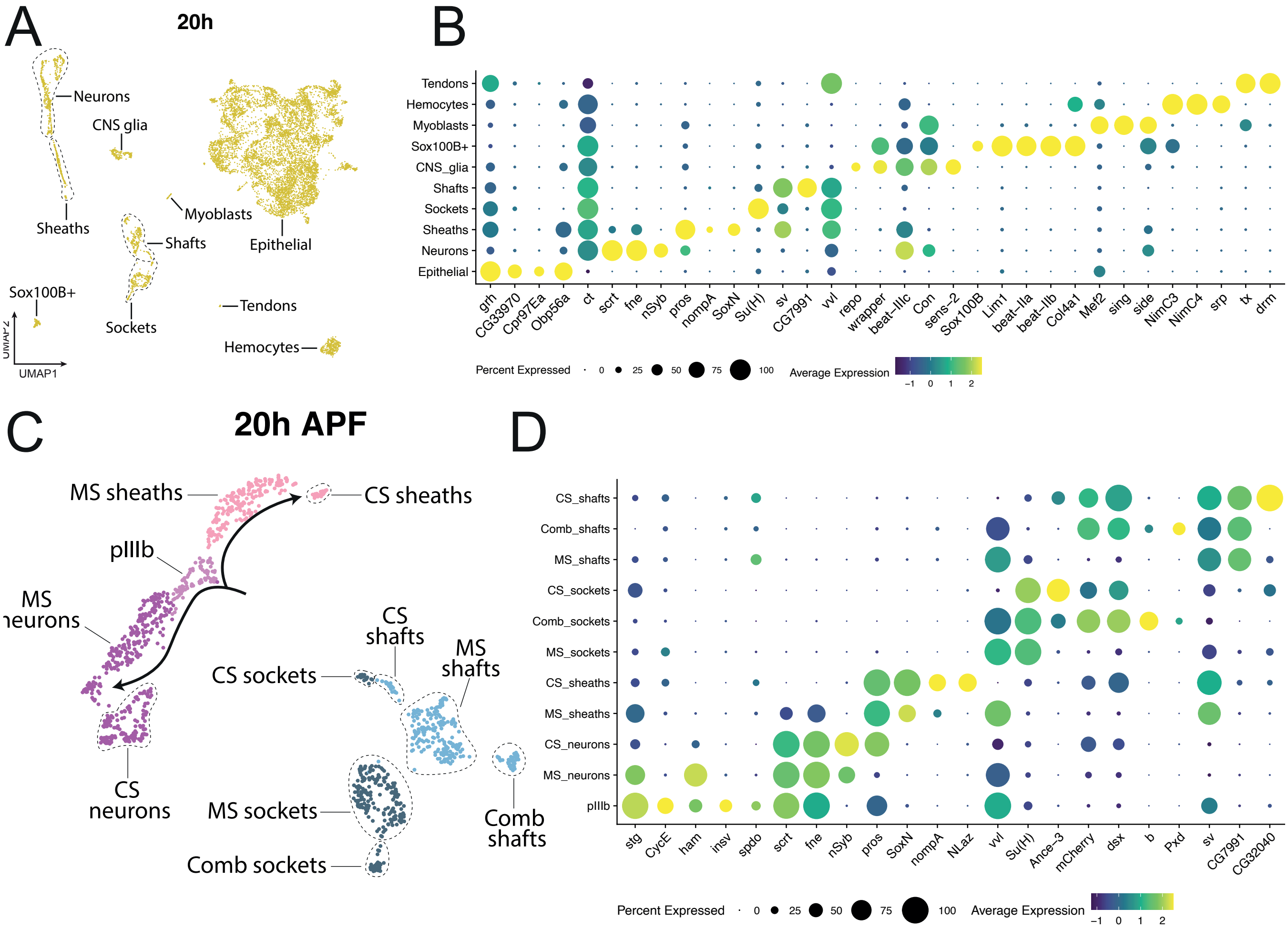

Supplementary figure 5. Identification of sensory cell populations in the 20h APF male dataset. (A) A UMAP of the 20h male dataset with major cell populations annotated. Annotation nomenclature follows that used in Hopkins *et al.* (81). (B) Genes enriched for expression in each annotated cluster labelled in (A). (C) The sensory organ lineage cells shown in (A), subsetted out and reclustered with cell types annotated. (D) Genes enriched for expression in each annotated cluster labelled in (C). Note that this dataset was generated from *dsx*-GAL4 > *UAS-mCherry* individuals, hence the inclusion of *mCherry* in the dotplot. MS=Mechanosensory, CS=Chemosensory. The data and code needed to generate the plots in this Figure can be found in https://osf.io/25bnc.
