# Supplementary material for: Metabolic and developmental rate divergence between serially homologous cells underlies an evolutionary innovation in Drosophila
Source: PLoS Biol. 2026 Aug 24;24(8):e3003964. doi: 10.1371/journal.pbio.3003964 (PMC13502693; doi:10.1371/journal.pbio.3003964)
Supplement: Supplementary file 7 [file pbio.3003964.s007.pdf]

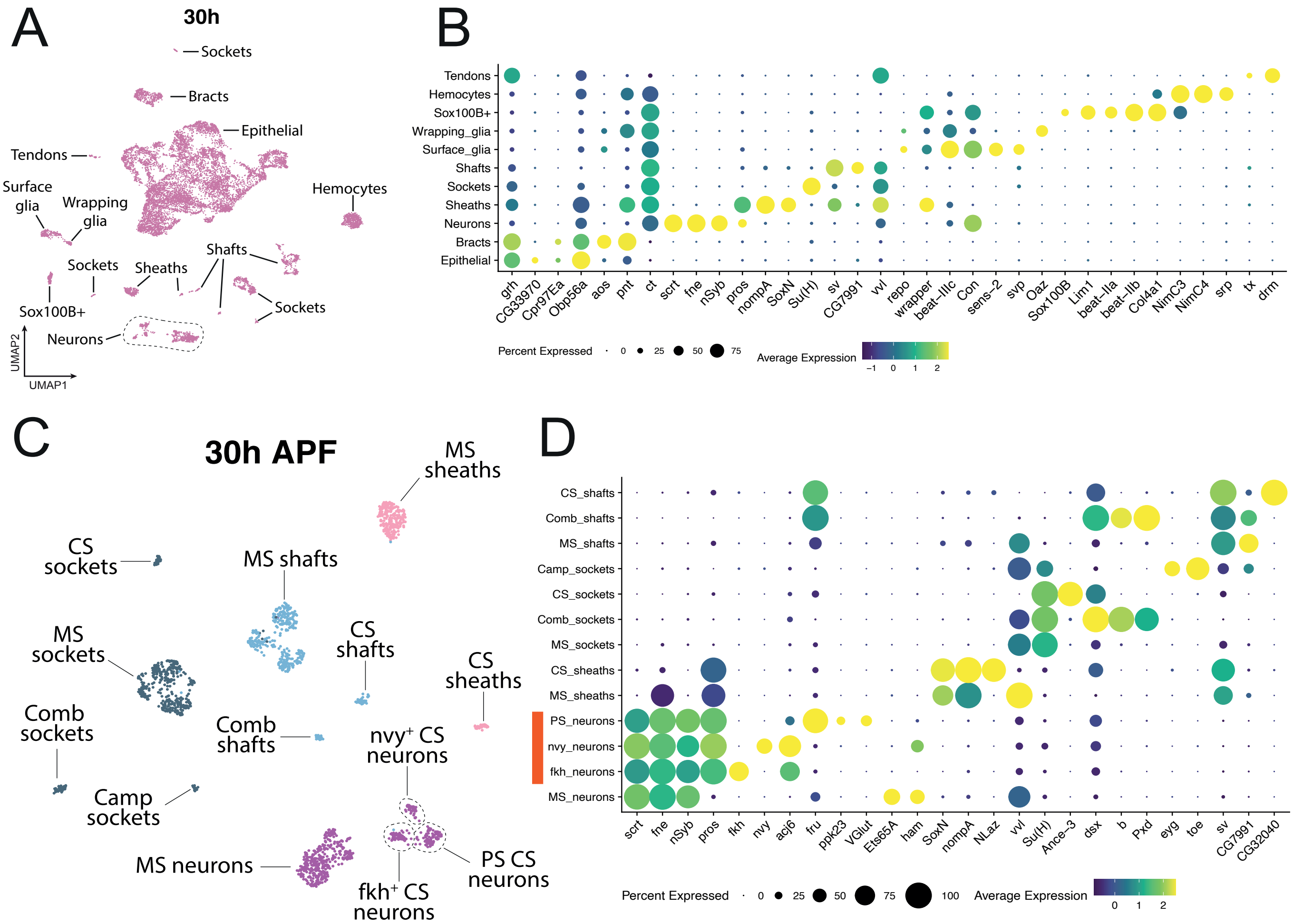

Supplementary figure 7. Identification of sensory cell populations in the 30h APF male dataset. (A) A UMAP of the 30h male dataset with major cell populations annotated. Annotation nomenclature follows that used in Hopkins *et al.* (81). (B) Genes enriched for expression in each annotated cluster labelled in (A). (C) The sensory organ lineage cells shown in (A), subsetted out and reclustered with cell types annotated. (D) Genes enriched for expression in each annotated cluster labelled in (C). Note that this dataset was generated from *RAL-517* individuals, so no fluorescent markers are shown. An orange bar denotes subpopulations of chemosensory neuron that were readily identifiable. nvy+ and fkh+ neurons follow the nomenclature used in Hopkins *et al.* (2023). PS neurons are the pheromone-sensing neurons, which at this clustering resolution could not readily be separated into the male- and female-sensing cells seen in Hopkins *et al.* MS=Mechanosensory, CS=Chemosensory, Camp.=Campaniform sensillum. The data and code needed to generate the plots in this Figure can be found in https://osf.io/25bnc.
