# Supplementary material for: Metabolic and developmental rate divergence between serially homologous cells underlies an evolutionary innovation in Drosophila
Source: PLoS Biol. 2026 Aug 24;24(8):e3003964. doi: 10.1371/journal.pbio.3003964 (PMC13502693; doi:10.1371/journal.pbio.3003964)
Supplement: Supplementary file 8 [file pbio.3003964.s008.pdf]

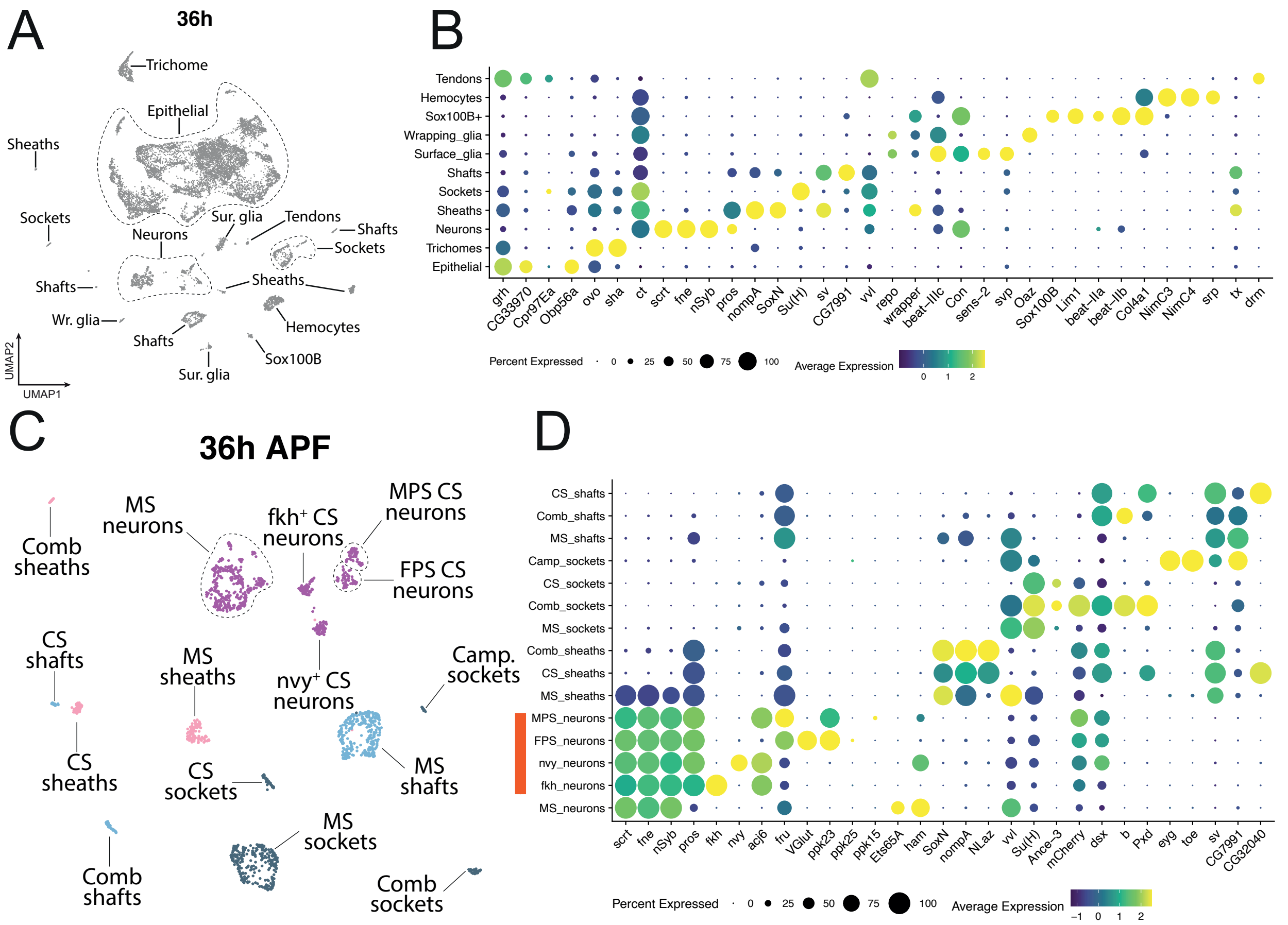


**Supplementary figure 8. Identification of sensory cell populations in the 36h APF male dataset.** (A) A UMAP of the 36h male dataset with major cell populations annotated. Annotation nomenclature follows that used in Hopkins *et al.* (81) with the new addition of trichome cells, which express ovo and sha. Wr. glia = Wrapping glia. Sur. glia = Surface glia. (B) Genes enriched for expression in each annotated cluster labelled in (A). (C) The sensory organ lineage cells shown in (A), subsetted out and reclustered with cell types annotated. (D) Genes enriched for expression in each annotated cluster labelled in (C). Note that this dataset was generated from *dsx*-GAL4 > *UAS-mCherry* individuals, hence the inclusion of *mCherry* in the dotplot. An orange bar denotes subpopulations of chemosensory neuron that were readily identifiable. nvy+, fkh+, male pheromone-sensing (MPS) and female pheromone-sensing (FPS) neurons follow the nomenclature used in Hopkins *et al.* MS=Mechanosensory, CS=Chemosensory, Camp.=Campaniform sensillum. The data and code needed to generate the plots in this Figure can be found in https://osf.io/25bnc.
