# Supplementary material for: Metabolic and developmental rate divergence between serially homologous cells underlies an evolutionary innovation in Drosophila
Source: PLoS Biol. 2026 Aug 24;24(8):e3003964. doi: 10.1371/journal.pbio.3003964 (PMC13502693; doi:10.1371/journal.pbio.3003964)
Supplement: Supplementary file 9 [file pbio.3003964.s009.pdf]

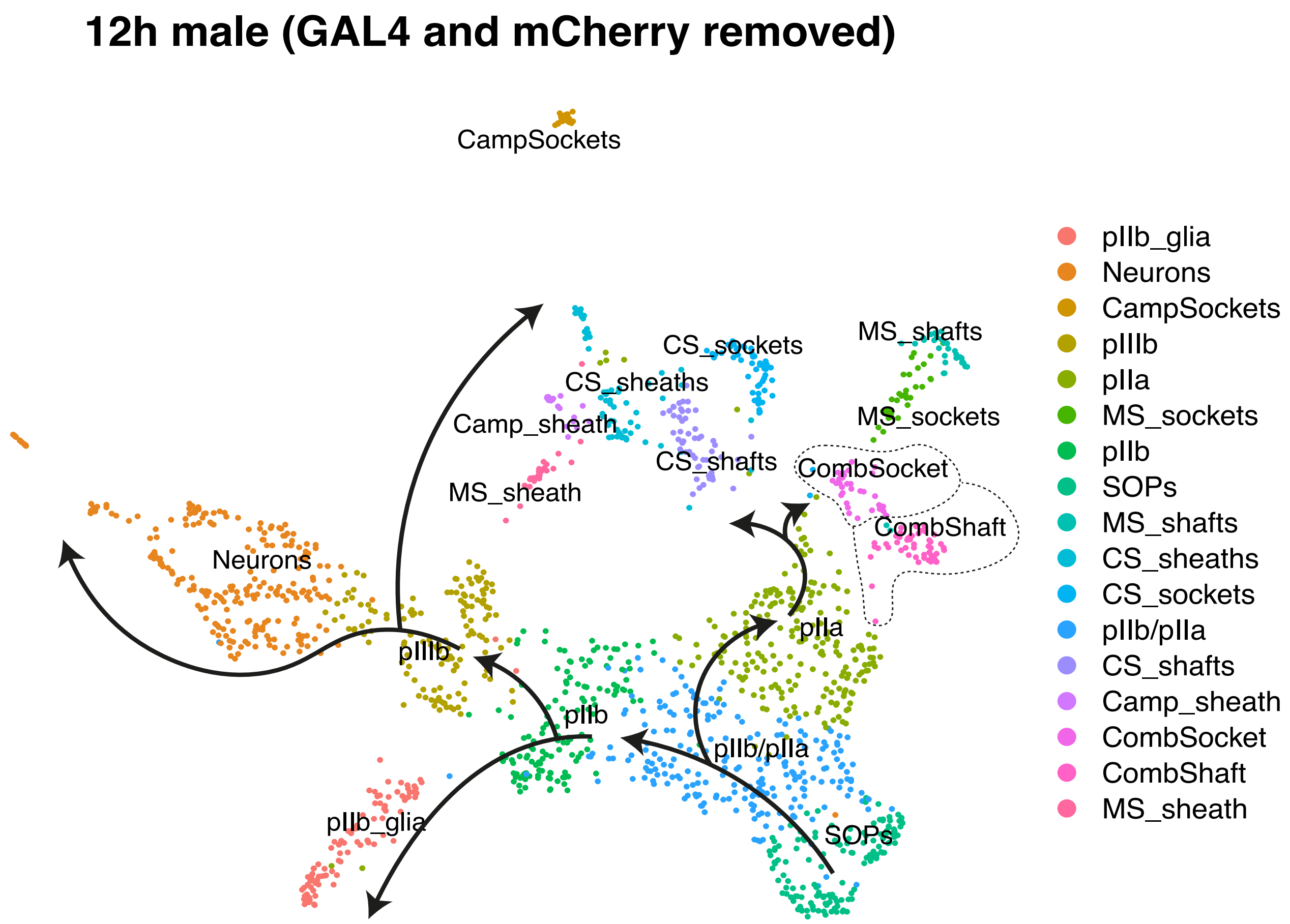

Supplementary figure 9. The distinct clustering of sex comb cells is not an artifact of the expression of Gal4 and mCherry. A UMAP of the sensory cells in the 12h APF male dataset after removing *Gal4* and *mCherry* and reclustering. Cells are coloured with the annotation they were given in the dataset that includes *Gal4* and *mCherry*. The comb socket and shaft populations still form distinct clusters indicating that their separation is a product of natural differences in expression. Arrows denote the differentiation trajectories. pIIIb cells divide to form neurons and sheaths, while pIIa cells divide to form sockets and shafts. The data and code needed to generate the plots in this Figure can be found in https://osf.io/25bnc.
