# Supplementary material for: Metabolic and developmental rate divergence between serially homologous cells underlies an evolutionary innovation in Drosophila
Source: PLoS Biol. 2026 Aug 24;24(8):e3003964. doi: 10.1371/journal.pbio.3003964 (PMC13502693; doi:10.1371/journal.pbio.3003964)
Supplement: Supplementary file 10 [file pbio.3003964.s010.pdf]

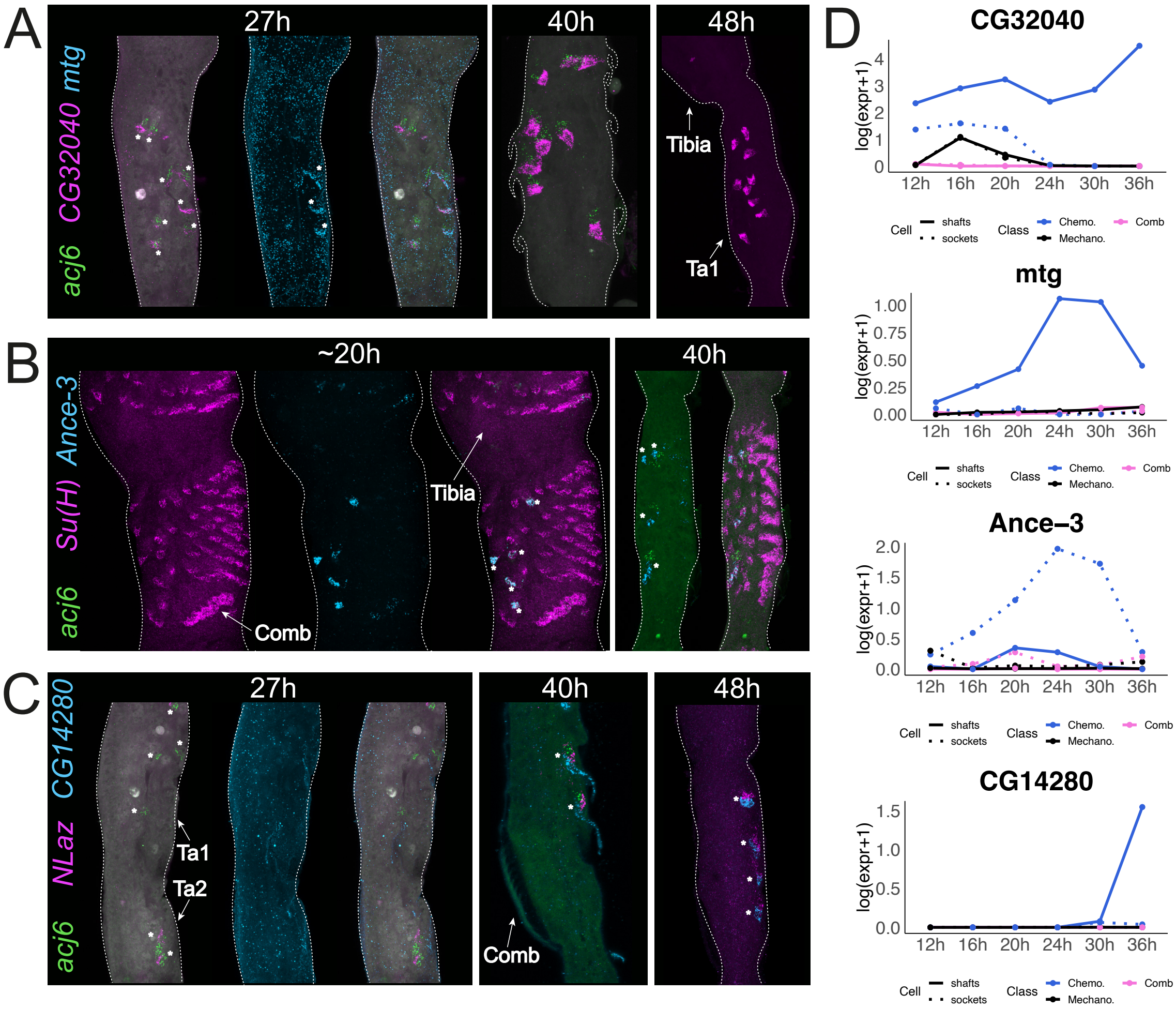

Supplementary figure 10. Accessory cells in chemosensory bristles express genes that are largely absent from their mechanosensory and sex comb homologues. (A-C) HCR in situ stainings against genes that are heavily enriched in chemosensory (CS) bristles relative to homologous cells in other bristle classes. *CG32040*, *mtg*, and *CG14280* in shafts, *Ance-3* in sockets, and *NLaz* in sheaths. We use *Su(H)* as a socket marker and *acj6* as a marker of 3 of the 4 neurons that innervate each CS bristle (81). The *mtg* probe shows high background staining throughout the leg but localized staining in the CS shaft is clear. Note that *mtg* staining in (A) is shown only at 27h APF and *acj6* staining in (C) is shown only at 27h APF and 40h APF. Asterisks denote the position of readily identifiable CS bristles in (B) and (C). (D) Time-series expression plots for genes largely specific to CS shaft and socket cells. The data and code needed to generate the plots in this Figure can be found in https://osf.io/25bnc.
