# Supplementary material for: Metabolic and developmental rate divergence between serially homologous cells underlies an evolutionary innovation in Drosophila
Source: PLoS Biol. 2026 Aug 24;24(8):e3003964. doi: 10.1371/journal.pbio.3003964 (PMC13502693; doi:10.1371/journal.pbio.3003964)
Supplement: Supplementary file 11 [file pbio.3003964.s011.pdf]

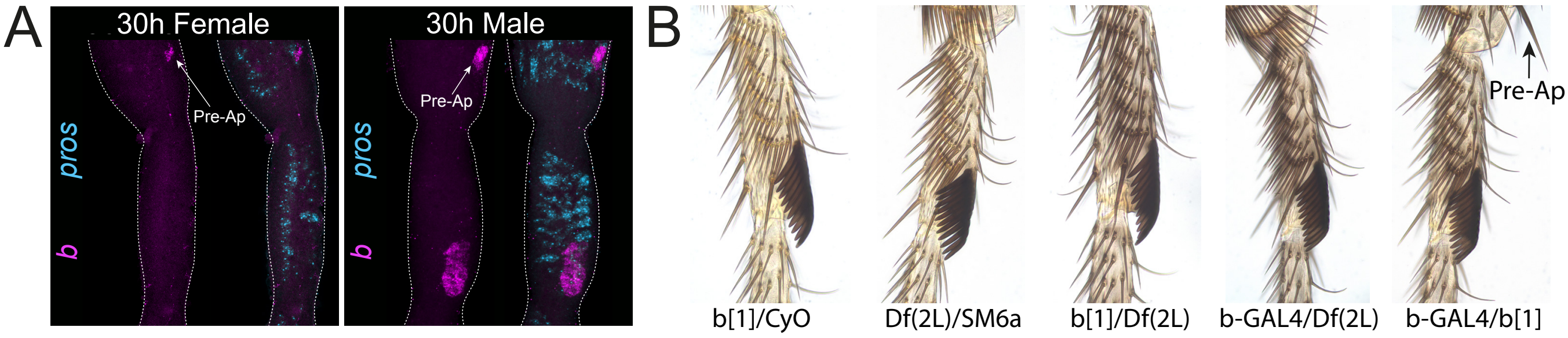

Supplementary figure 11. *black* is specific to the sex comb in the first tarsal segment but is not required for normal sex comb development. (A) HCR in situ staining of *b* and *pros* at 30h APF in a male and female first tarsal segment. *pros* is used here as a bristle marker, being expressed in the sheath cell of every bristle and the neurons of chemosensory bristles (81). The position of the sex comb is labeled, as is the likely position of the pre-apical ('pre-ap') bristle in the distal tibia. (B) First tarsal segments from adult males that are transheterozygous for mutant alleles affecting *b*. These alleles are a molecular null (*b[1]*) (82), a hypomorphic GAL4 line (*b-GAL4*)(86), and a deficiency that covers the *b* locus on chromosome arm 2L (*Df(2L)*). Genotypes heterozygous for the molecular null or deficiency line over either the *CyO* or *SM6a* balancer are shown as controls. The pre-apical bristle in the distal tibia is labelled in one image. Note how transheterozygotes show the darker cuticular melanization phenotype known to be associated with *b* nulls (82) but no obvious defects in the sex comb.
