# Supplementary material for: Metabolic and developmental rate divergence between serially homologous cells underlies an evolutionary innovation in Drosophila
Source: PLoS Biol. 2026 Aug 24;24(8):e3003964. doi: 10.1371/journal.pbio.3003964 (PMC13502693; doi:10.1371/journal.pbio.3003964)
Supplement: Supplementary file 12 [file pbio.3003964.s012.pdf]

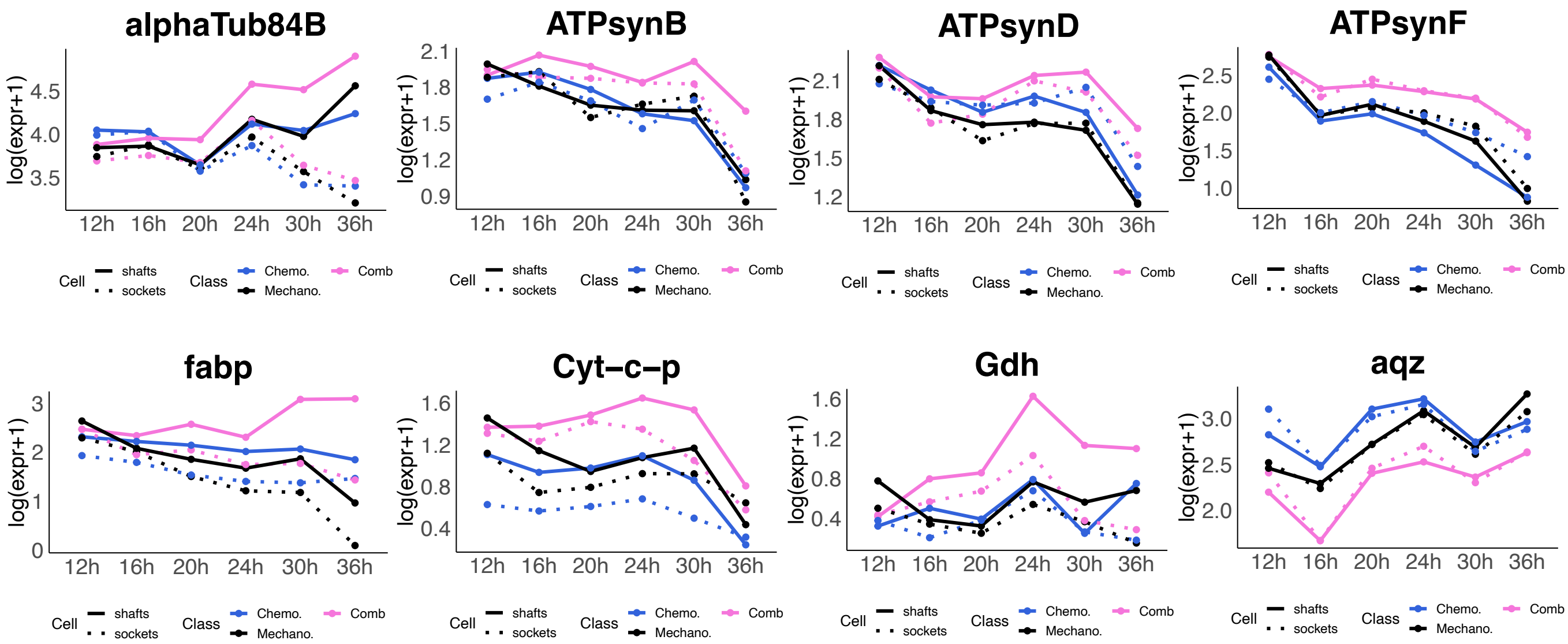

Supplementary figure 12. Quantitative upregulation of genes in the sex comb and chemosensory bristles. Time-series expression plots for genes showing significant quantitative expression differences between sex comb and mechanosensory bristle cells, without evidence of heterochrony. The exception is *aqz*, which shows quantitative upregulation in chemosensory rather than sex comb cells. The data and code needed to generate the plots in this Figure can be found in https://osf.io/25bnc.
