# Supplementary material for: Metabolic and developmental rate divergence between serially homologous cells underlies an evolutionary innovation in Drosophila
Source: PLoS Biol. 2026 Aug 24;24(8):e3003964. doi: 10.1371/journal.pbio.3003964 (PMC13502693; doi:10.1371/journal.pbio.3003964)
Supplement: Supplementary file 13 [file pbio.3003964.s013.pdf]

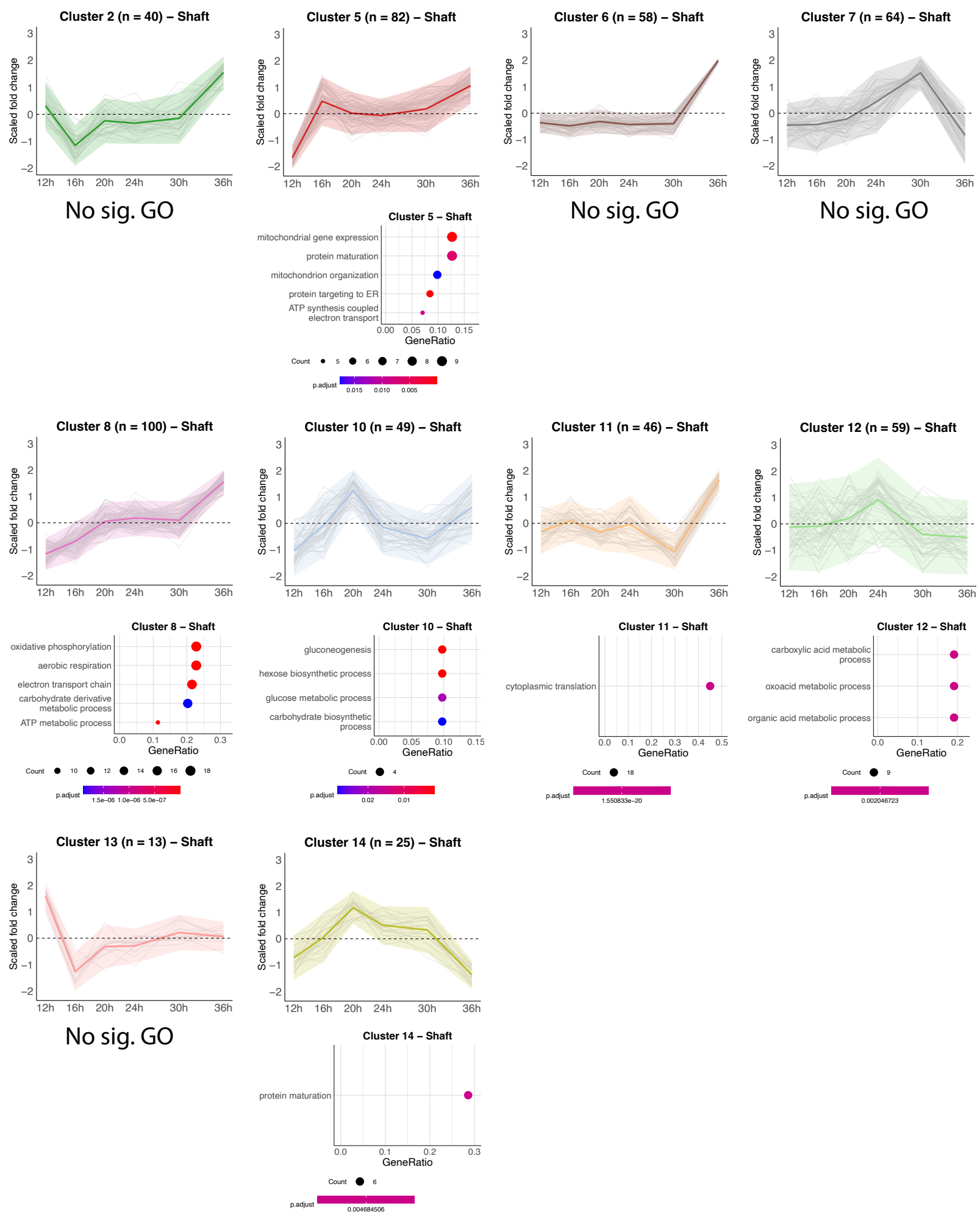

Supplementary figure 13. Expression trajectories for genes found to be differentially expressed between sex comb shafts and their mechanosensory homologs. All clusters that are not present in Figure 4 are shown. Genes are grouped based on the cluster they were assigned to in our DPGP analysis. Each expression plot shows the trajectory of each gene in the cluster (gray lines) as well as the cluster mean (thick, colored line). The ribbon denotes ± 2 S.D. The number of genes in the cluster is given in the plot title. The values plotted are the log2 fold changes between sex comb and mechanosensory (MS) cells normalized by Z-score transformation (as in 35). Below each plot is a dot plot showing a selection of up to 5 biological process GO terms found to be significantly enriched in each cluster relative to a background of all the genes detected in MS and comb shaft cells at one timepoint or more. The data and code needed to generate the plots in this Figure can be found in https://osf.io/25bnc.
