# Supplementary material for: Metabolic and developmental rate divergence between serially homologous cells underlies an evolutionary innovation in Drosophila
Source: PLoS Biol. 2026 Aug 24;24(8):e3003964. doi: 10.1371/journal.pbio.3003964 (PMC13502693; doi:10.1371/journal.pbio.3003964)
Supplement: Supplementary file 14 [file pbio.3003964.s014.pdf]

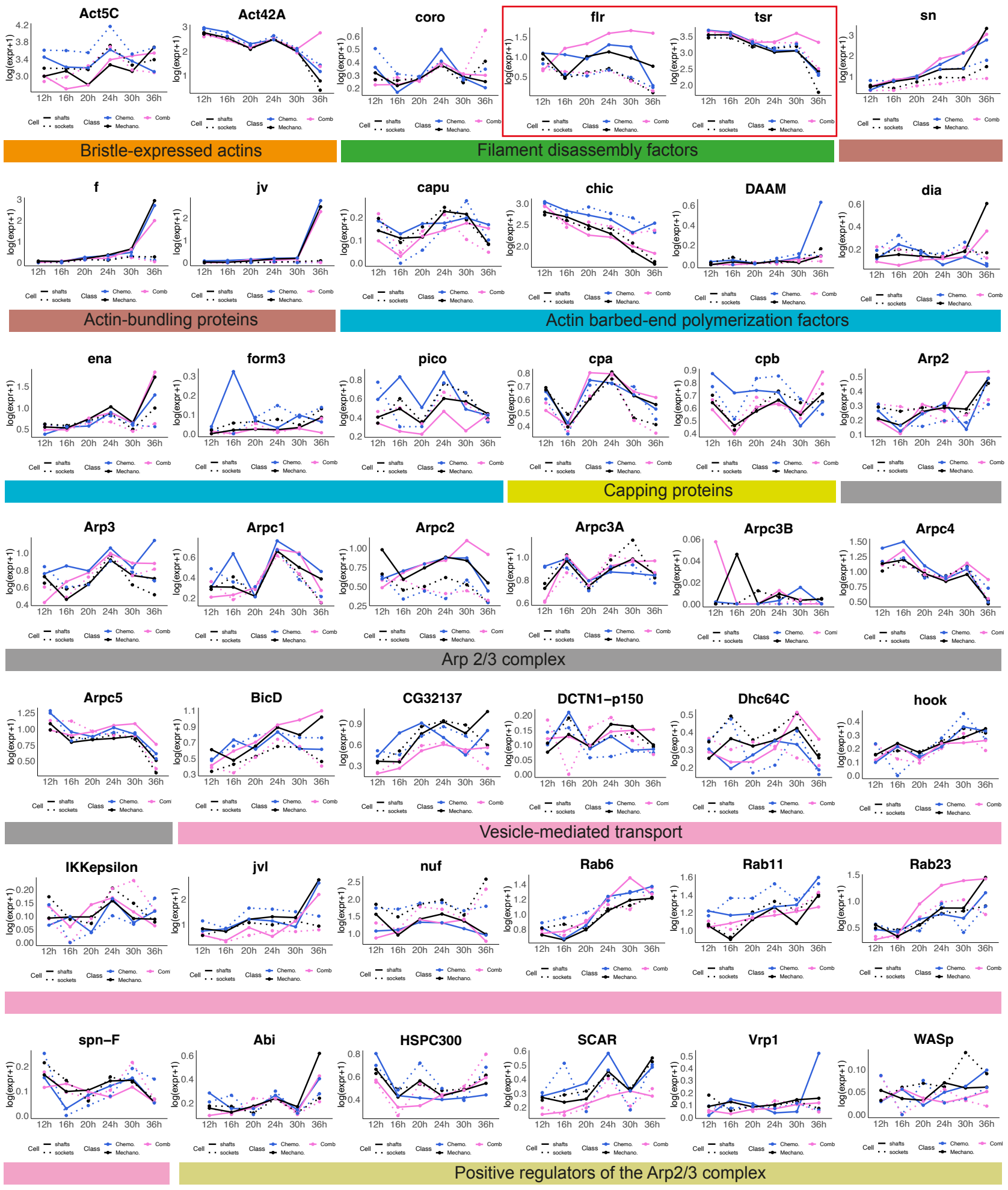

Supplementary figure 14. Sex comb shafts modulate gene networks that regulate actin filament depolymerization. Time-series gene expression plots, grouped by the actin-related function each gene performs. Sex comb-enriched expression of the filament disassembly factors *flr* and *tsr* (highlighted with red boxes) provide a potential route through which shaft growth can be accelerated. By pruning and disassembling filaments that are not bundled, these factors ensure the continued availability of actin monomers at the growing tip (38). Note that in many cases, there is no clear difference between shafts and sockets, let alone different classes of shaft. The data and code needed to generate the plots in this Figure can be found in https://osf.io/25bnc.
