# Supplementary material for: Metabolic and developmental rate divergence between serially homologous cells underlies an evolutionary innovation in Drosophila
Source: PLoS Biol. 2026 Aug 24;24(8):e3003964. doi: 10.1371/journal.pbio.3003964 (PMC13502693; doi:10.1371/journal.pbio.3003964)
Supplement: Supplementary file 16 [file pbio.3003964.s016.pdf]

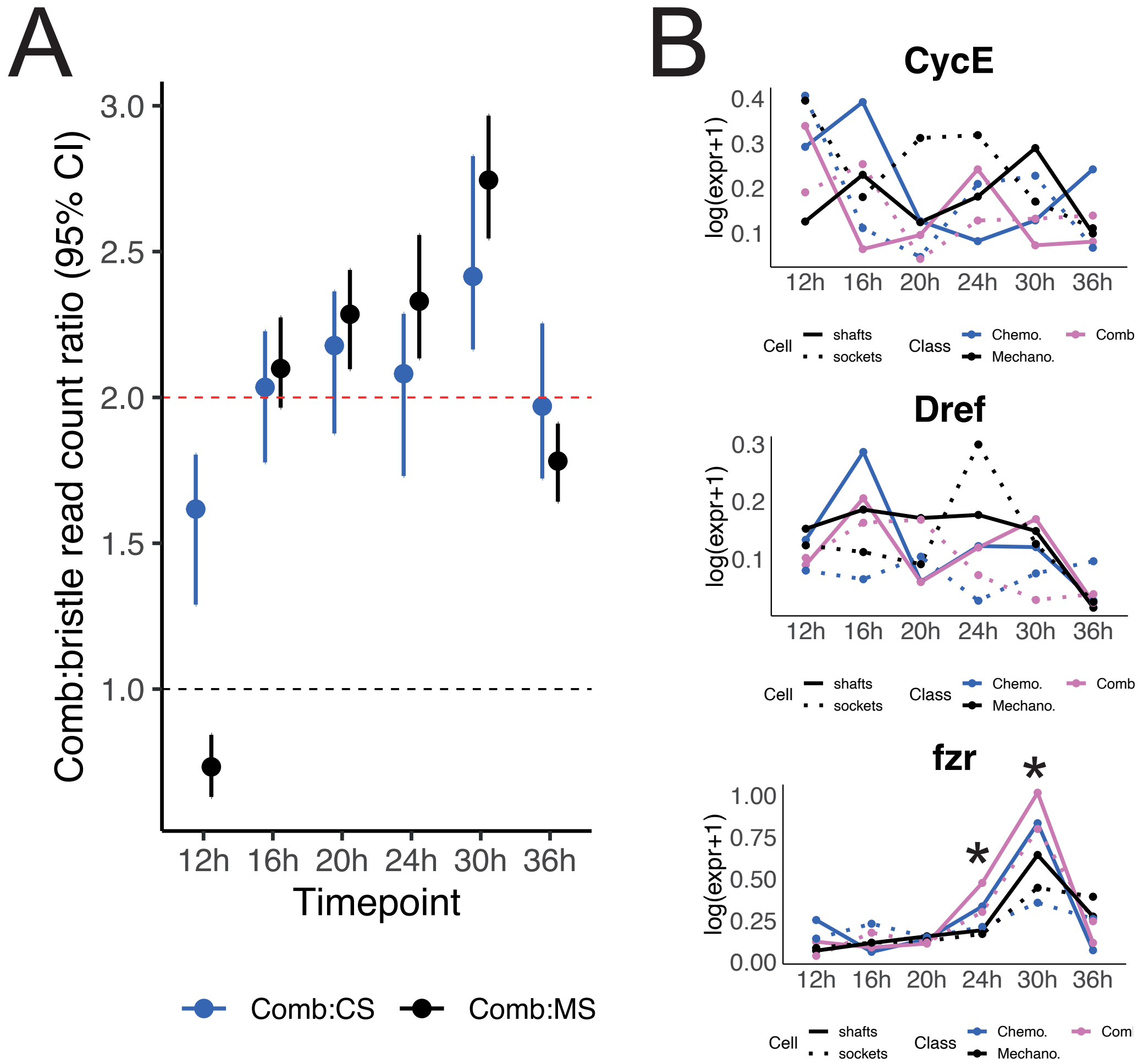

Supplementary figure 16. Changes in endoreplication dynamics are a key part of the bristle-to-tooth transformation. (A) The ratio of sex comb shaft to mechanosensory (MS) shaft and chemosensory (CS) shaft read counts at each timepoint. A value of 1 suggests equivalent read counts between, for instance, MS shaft and comb shaft cells, while a value of 2 means that we detect twice as many reads in comb cells. Bootstrapped confidence intervals (2000 iterations) for the ratios are presented. (B) Time-series expression plots for endoreplication-mediating genes we tested for roles in sex comb development using the UAS/GAL4 system. Asterisks denote timepoints where a gene was significantly upregulated in sex comb shafts relative to mechanosensory shafts. Of the three genes we tested—*Dref*, *CycE*, and *fzr*—only *fzr* was significantly upregulated in sex comb cells relative to MS bristles at any time point in our scRNA-seq data, raising the possibility that it might be a direct regulator of the ploidy differences between sex comb and mechanosensory cells. However, we did not see a corresponding increase in MS bristles when overexpressing *fzr* under the control of the bristle driver *Pax2*-GAL4, suggesting that *fzr* alone is insufficient to drive the additional endocycles seen in sex comb cells. The data and code needed to generate the plots in this Figure can be found in https://osf.io/25bnc.
