# Supplementary material for: Metabolic and developmental rate divergence between serially homologous cells underlies an evolutionary innovation in Drosophila
Source: PLoS Biol. 2026 Aug 24;24(8):e3003964. doi: 10.1371/journal.pbio.3003964 (PMC13502693; doi:10.1371/journal.pbio.3003964)
Supplement: Supplementary file 17 [file pbio.3003964.s017.pdf]

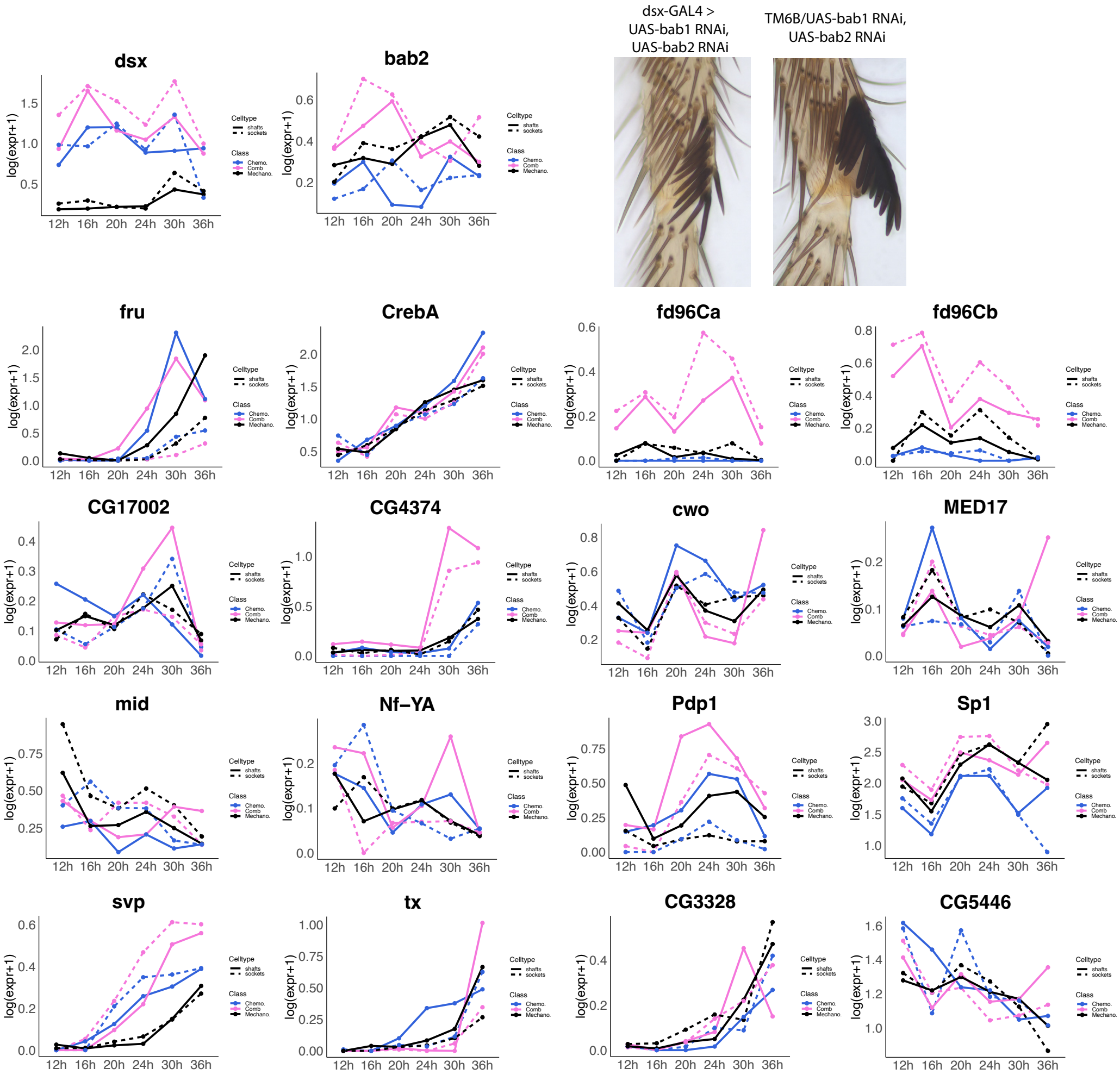

Supplementary figure 17. Transcription factor knockdowns in the developing sex comb suggest a decentralized genetic pathway. Expression plots for each transcription factor that we detected as significantly upregulated in sex comb cells at any timepoint. With the exception of *dsx* and the forkhead domain genes *fd96Ca* and *fd96Cb* (which have been tested previously, 51), we used UAS-RNAi constructs to knockdown the expression of each transcription factor in the developing sex comb under the control of *dsx*-GAL4. Only a double knockdown of *bab1* and *bab2* (87) gave a phenotype, showing an incomplete transformation of sex comb teeth towards the thinner, shorter proportions of a mechanosensory bristle. The data and code needed to generate the plots in this Figure can be found in https://osf.io/25bnc.
