# Supplementary material for: Metabolic and developmental rate divergence between serially homologous cells underlies an evolutionary innovation in Drosophila
Source: PLoS Biol. 2026 Aug 24;24(8):e3003964. doi: 10.1371/journal.pbio.3003964 (PMC13502693; doi:10.1371/journal.pbio.3003964)
Supplement: Supplementary file 18 [file pbio.3003964.s018.pdf]

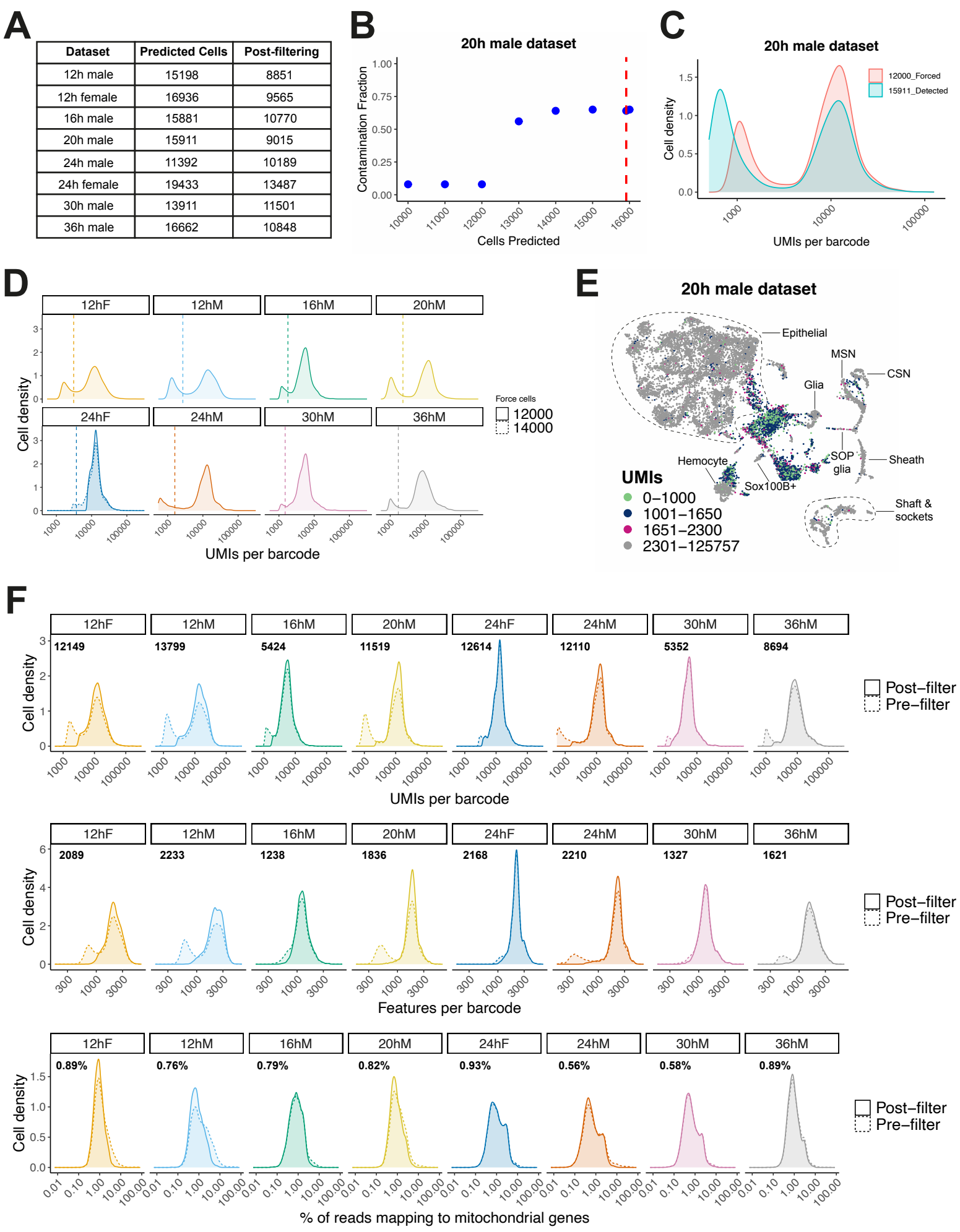

Dataset	Predicted Cells	Post-filtering
12h male	15198	8851
12h female	16936	9565
16h male	15881	10770
20h male	15911	9015
24h male	11392	10189
24h female	19433	13487
30h male	13911	11501
36h male	16662	10848

Supplementary figure 18. Processing the single-cell data. (A) The number of predicted cells recovered in each dataset when running CellRanger without ‘force-cells’, alongside the final number of cells in each dataset after filtering cells out based on dataset-specific quality control thresholds. (B) The contamination fraction estimated in the 20h APF dataset when forcing CellRanger to detect set numbers of cells. The dashed red line indicates the number of cells detected (15,911) using default settings (i.e., without force-cells). (C) The distribution of UMIs detected per barcode in the 20h dataset when using default settings or forcing the detection of 12,000 cells. Note the reduction in low barcode ‘cells’ (i.e., putative empty droplets) when instructing CellRanger to force the detection of fewer cells. (D) The distribution of UMIs detected per barcode in each dataset when forcing the detection of 12,000 cells. Note that the female 24h dataset also includes the distribution when forcing the detection at 14,000 cells. We used a higher value for this dataset because cutting at 12,000 lost some of the lower part of the distribution that was retained in all other datasets. (E) A UMAP of the full, force-cells, 20h dataset without any downstream filtering of cells based on quality control metrics. Note how low UMI cells generally form their own cluster or fall between closely associated clusters in UMAP space (e.g., between chemosensory and mechanosensory neuron clusters), suggestive of their aberrant characteristics. (F) The distribution of UMIs, features, and percentage of reads mapping to mitochondrial genes for each of the datasets pre- and post-filtering based on cell-level quality control metrics. The post-filtering median value is provided as a label in each case. The data and code needed to generate the plots in this Figure can be found in https://osf.io/25bnc.
